# Characterization of TNF-induced cell death in *Drosophila* reveals caspase- and JNK-dependent necrosis and its role in tumor suppression

**DOI:** 10.1038/s41419-019-1862-0

**Published:** 2019-08-14

**Authors:** Mingli Li, Shiyao Sun, Jessica Priest, Xiaolin Bi, Yun Fan

**Affiliations:** 1 School of Biosciences, University of Birmingham, Birmingham, UK; 2 College of Basic Medical Sciences, Dalian Medical University, Dalian, China

**Keywords:** Necroptosis, Disease model

## Abstract

Tumor-necrosis factor (TNF) and its superfamily members are pleiotropic cytokines. Activation of TNF can lead to distinct cellular outcomes including inflammation, cell survival, and different forms of cell death, such as apoptosis and necrosis in a context-dependent manner. However, our understanding of what determines the versatile functions of TNF is far from complete. Here, we examined the molecular mechanisms that distinguish the forms of cell death induced by Eiger (Egr), the sole homolog of TNF in *Drosophila*. We show that expression of Egr in the developing *Drosophila* eye simultaneously induces apoptosis and apoptosis-independent developmental defects indicated by cellular disorganization, both of which rely on the c-Jun N-terminal kinase (JNK) signaling activity. Intriguingly, when effector caspases DrICE and Dcp-1 are defective or inhibited, expression of Egr triggers necrosis which is characterized by loss of cell membrane integrity, translucent cytoplasm, and aggregation of cellular organelles. Moreover, such Egr-induced necrosis depends on the catalytic activity of the initiator caspase Dronc and the input from JNK signaling but is independent of their roles in apoptosis. Further mosaic analysis with mutants of *scribble* (*scrib*), an evolutionarily conserved tumor suppressor gene regulating cell polarity, suggests that Egr/JNK-mediated apoptosis and necrosis establish a two-layered defense system to inhibit the oncogenic growth of *scrib* mutant cells. Together, we have identified caspase- and JNK-dependent mechanisms underlying Egr-induced apoptosis versus necrosis and their fail-safe roles in tumor suppression in an intact organism in vivo.

## Introduction

Apoptosis is a major form of programmed cell death critical for development and damage response^1^. The key factors driving apoptosis are caspases, a family of cysteine proteases. These apoptotic caspases can further group into initiator and effector caspases^2^. The initiator caspases, once activated, cleave and activate the effector caspases leading to apoptosis. Unlike apoptosis, necrosis has long been considered as an uncontrolled form of cell death. However, recent studies have revealed that certain types of necrosis are molecularly regulated^3,4^. Intriguingly, our growing understanding of apoptosis and regulated necrosis has unveiled multiple molecular interplays between them^5^. One example of such molecules is the tumor-necrosis factor (TNF) superfamily, a group of cytokines which was initially discovered because of its antitumor activity^6^. Binding of TNF to their receptors promotes recruitment of various death-inducing protein complexes depending on the context^7^. A key component common in these complexes is caspase-8, an initiator caspase. Once activated, caspase-8 cleaves and activates effector caspases, such as caspase-3 and −7, to trigger apoptosis^8^. Notably, caspase-8 also cleaves and inactivates two receptor-interacting serine/threonine-protein kinases (RIPKs), RIPK1, and RIPK3, which mediate activation of necroptosis, a form of regulated necrosis^9–12^. Therefore, when Caspase-8 is deficient or inhibited, activation of TNF induces necrosis via RIPK1 and PIPK3.

In addition to cell death, functions of TNF family members have been revealed in immunity, inflammation, cell survival, and proliferation^6^. Many of these functions are mediated by pleiotropic molecules, such as the Nuclear Factor-κB (NF-κB) transcription factor^13^, the c-Jun N-terminal kinase (JNK), and the p38 mitogen-activated protein kinase (p38-MAPK)^14,15^. It is therefore not surprising that dysregulated TNF signaling is associated with numerous pathological conditions including cancer^16,17^. However, our understanding of the mechanisms that determine distinct outcomes of TNF signaling is still far from complete.

Eiger (Egr) is the sole *Drosophila* homolog of TNF^18,19^. Wengen^20^ and, more recently, Grindelwald^21^ have been identified as its receptors. Similar to mammalian TNFs, Egr plays multiple roles in regulating cell death, host defense, tissue growth, and regeneration in a context-dependent manner^22–24^. Notably, Egr exerts its functions mainly through activation of JNK. For example, expression of Egr under the control of an eye-specific driver GMR (*GMR* > *egr*) activates JNK leading to cell death and small adult eyes^18,19,25^. Intriguingly, *GMR* > *egr*-induced cell death may be nonapoptotic because its eye ablation phenotype cannot be suppressed by P35 (ref.^18^), an inhibitor of the effector caspases DrICE and Dcp-1 (ref.^26^). However, what seems to be a paradox, *GMR* > *egr*-induced small eyes can be suppressed by inhibition of the initiator caspase Dronc^19,25^. It is therefore not yet clear whether and how Egr can induce both apoptotic and nonapoptotic cell death.

Here, we report that Egr primarily induces JNK-dependent apoptosis and, simultaneously, apoptosis-independent cellular disorganization when it is expressed in the *Drosophila* eye. However, when apoptosis is blocked by inhibition of the effector caspases DrICE and Dcp-1, expression of Egr induces necrosis instead. Intriguingly, the initiator caspase Dronc is required for both Egr-induced apoptosis and necrosis, but at different protein levels. Its catalytic activity cooperates with an apoptosis-independent role of JNK signaling to activate necrosis. Furthermore, under pathological conditions, e.g., in cells mutant for *scribble* (*scrib*) which is a tumor suppressor gene regulating cell polarity, activation of Egr-JNK establishes a two-layered defense mechanism with apoptosis and, if apoptosis fails, necrosis in place to prevent oncogenic tissue overgrowth. Therefore, *Drosophila* models can be employed to dissect the molecular interplays between apoptosis and necrosis in vivo.

## Results

### The cleaved Dcp-1 antibody is a specific marker for activated effector caspases DrICE and Dcp-1 in *Drosophila*

**Fig. 1:**
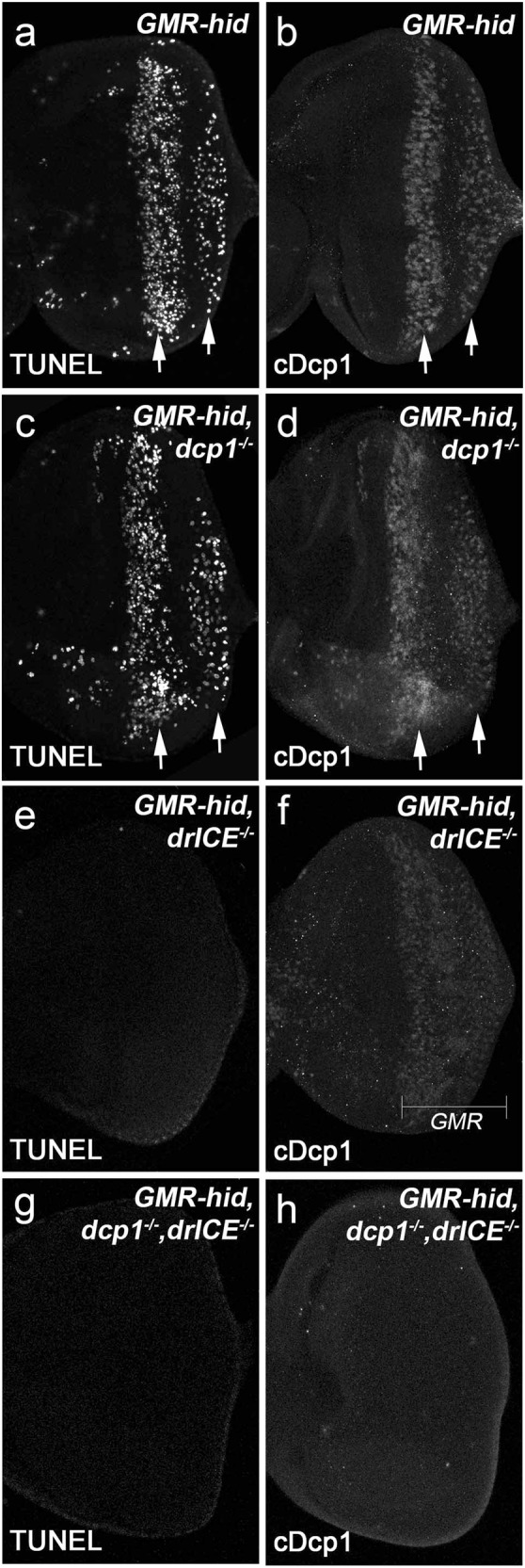
The cDcp1 antibody recognizes cleaved DrICE and Dcp-1 in *Drosophila*. Late 3rd instar larval eye disks labeled with either the TUNEL assay (**a**, **c**, **e**, **g**) or the cDcp1 antibodies (**b**, **d**, **f**, **h**). Expression of *hid* under the control of *GMR* (*GMR-hid*) induces two apoptotic waives indicated by either TUNEL (**a**, arrows) or cDcp1 (**b**, arrows) staining. *GMR-hid*-induced two apoptotic waves are recognized by both TUNEL (**c**) and cDcp1 (**d**) in *dcp-1* null mutants. *GMR-hid*-induced apoptosis is blocked in *drICE* null mutants as indicated by lack of TUNEL labeling (**e**). However, cDcp1 recognizes a low level of signals in the whole *GMR* domain in the same genetic background (**f**). No *GMR-hid*-induced signals were detected by either TUNEL (**g**) or cDcp1 (**h**) in *drICE* and *dcp-1* double null mutants

**Fig. 2:**
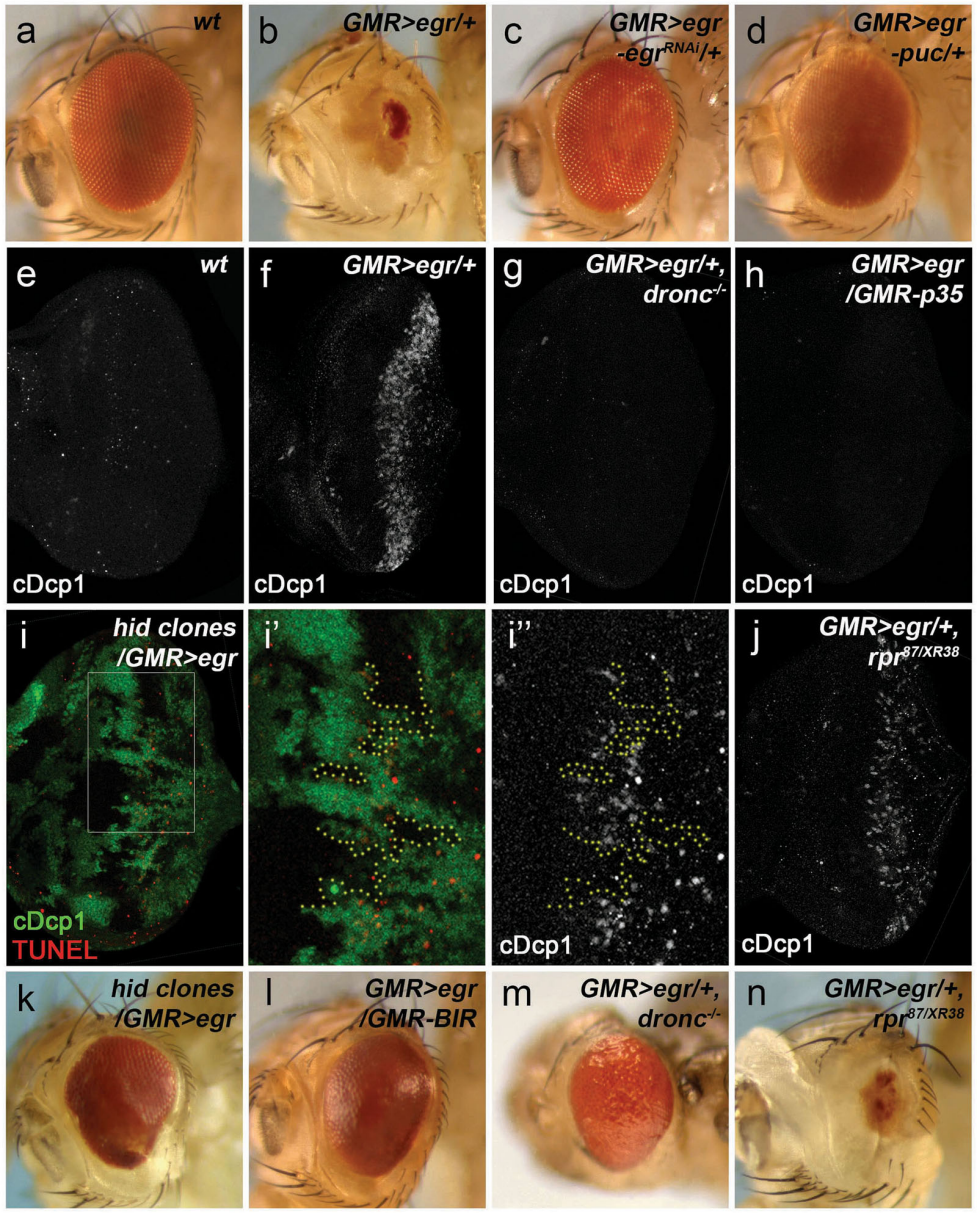
*GMR* > egr induces apoptosis via hid in the *Drosophila* eye. **a**–**d** Adult eye images. Compared with wild type (**a**), expression of *egr* under the control of *GMR* (*GMR-GAL4 UAS-egr, GMR* > *egr*) induces a strong eye ablation phenotype (**b**). This phenotype is completely suppressed by an RNAi knockdown of *egr* (*egr*^*RNAi*^, **c**) or expression of *puckered* (*puc*, **d**), a negative regulator of JNK. Late 3rd instar larval eye disks labeled with cDcp1 (**e**–**h**, **j**) or cDcp1 and GFP (**i**–**i′′**). Compared with wild type (**e**), *GMR* > *egr* induces massive apoptosis indicated by cDcp1 labeling (**f**). This apoptosis is suppressed in *dronc* null mutants (**g**) or by expression of a *GMR-p35* transgene (**h**). In *GMR* *>* *egr* disks with *hid* mutant clones marked by lack of GFP (**i**), apoptosis is blocked in the clones (highlighted by yellow dotted lines in **i′** and **i**′′ which are enlarged images of the outlined area in **i**). In contrast, *rpr* mutants (*rpr*^*87/XR38*a^, combination of a deletion and a null mutant of *rpr*) do not suppress *GMR* > *egr*-induced apoptosis (**j**). **k**–**n** Adult eye images. *hid* mutant clones (**k**), expression of a RING domain-deleted, therefore stabilized, form of Diap1 (*GMR-BIR*, **l**) or *dronc* null mutants (pharate adults were dissected out of the pupal cases, **m**) strongly suppress *GMR* > *egr*-induced eye ablation phenotype. In contrast, *rpr* mutants (*rpr*^*87/XR38*a^) do not suppress the small eyes induced by *GMR* > *egr* (**n**)

To determine whether *GMR* > *Egr* induces apoptosis or nonapoptotic cell death, we first sought to identify a marker specifically recognizing activated effector caspases, e.g., the cleaved DrICE and Dcp-1, in *Drosophila* because antibodies recognizing the cleaved human caspase-3 are not specific to these proteins^27^. A recently developed cleaved Dcp-1 (Asp216) antibody (referred to as cDcp1) from Cell Signaling Technology is a polyclonal antibody raised against the large 22 kDa fragment of cleaved Dcp-1. Although this antibody has been increasingly used to label apoptosis in *Drosophila*^28^, a detailed characterization of its specificity has not been performed. To address this, we used *GMR-hid*, a transgene activating apoptosis in the eye^29^. In late 3rd instar larval eye disks, compared with wild type (Fig. 2e), *GMR-hid* induces two waves of apoptotic cells as indicated by TUNEL, an assay detecting DNA fragmentation therefore labeling apoptotic cells (Fig. 1a)^30^. The cDcp1 antibodies recognize these two apoptotic waves (Fig. 1b). Interestingly, these apoptotic signals persist in *dcp-1* null mutants (Fig. 1c, d). It suggests that cleaved Dcp-1 is not the only protein recognized by cDcp1. In addition to Dcp-1, DrICE is another major effector caspase in somatic tissues^31,32^. Indeed, *GMR-hid*-induced apoptosis is almost completely lost in *drICE* null mutants (Fig. 1e). Because DrICE and Dcp-1 share similar sequences cleaved by Dronc^27,33^, it is possible that cDcp1 also recognizes the cleaved DrICE. Consistent with this idea, cDcp1 detects a relatively low level of proteins, presumably the cleaved Dcp-1, in *drICE* mutants (Fig. 1f). In contrast, the cDcp1- and TUNEL-signals are lost in *dcp-1; drICE* double mutants (Fig. 1g, h). Therefore, cDcp1 recognizes both cleaved Dcp-1 and cleaved DrICE.

### Expression of Eiger induces strong apoptosis through the canonical apoptosis pathway and the pro-apoptotic gene *hid*

It has been reported that *GMR* > *egr* activates JNK resulting in an eye ablation phenotype (compare 2b to 2a)^18,19^, which can be suppressed by RNAi knockdown of *egr* (Fig. 2c) or expression of *puckered* (*puc*) (Fig. 2d), a negative regulator of JNK^34,35^. To assess whether *GMR* > *egr* induces apoptosis, we used the cDcp1 antibody. Compared with wild type (Fig. 2e), a strong wave of cDcp1-labeling was observed in *GMR* > *egr* disks (Fig. 2f). To confirm it is apoptosis, we performed genetic analyses on the key components in the apoptosis pathway. Loss of Dronc, the major initiator caspase mediating apoptosis in *Drosophila*^36^, or expression of P35, an inhibitor of activated DrICE and Dcp-1 (ref.^26^), completely blocks the cDcp1 signals in *GMR* > *egr* (Fig. 2g, h). Consistently, *GMR* > *egr*-induced eye ablation phenotype is suppressed in *dronc* null mutants (compare Fig. 2m to 2b). Moreover, expression of a RING domain-deleted (therefore stabilized) form of Diap1 (BIR), an apoptosis inhibitor acting upstream of Dronc^37^, also suppresses *GMR* > *egr* small eyes (compare Fig. 2l to 2b). Therefore, *GMR* > *egr* triggers massive apoptosis in the developing *Drosophila* eye.

To identify which proapoptotic genes mediate *GMR* > *egr*-induced apoptosis, we examined expression of *hid* and *rpr* by using their reporters^38^. Compared with the control, expression of *hid*, but not *rpr*, significantly increases in *GMR* *>* *egr* (Supplementary Fig. S1). Moreover, loss of *rpr* in *rpr87/*XR38, a combination of a deletion^39^ and a null mutant of *rpr* (ref.^40^), does not affect *GMR* > *egr*-induced apoptosis (Fig. 2j). In contrast, such apoptosis is lost in *hid* mutant clones (Fig. 2i–i′′). Consistent with these results, the *hid* mutant clones, but not *rpr* mutants, suppress *GMR* > *egr*-induced small eyes (Fig. 2k, n). Taken together, expression of Egr activates apoptosis through the proapoptotic gene *hid* in the developing *Drosophila* eye.

### Expression of Eiger induces developmental defects independent of apoptosis

**Fig. 3:**
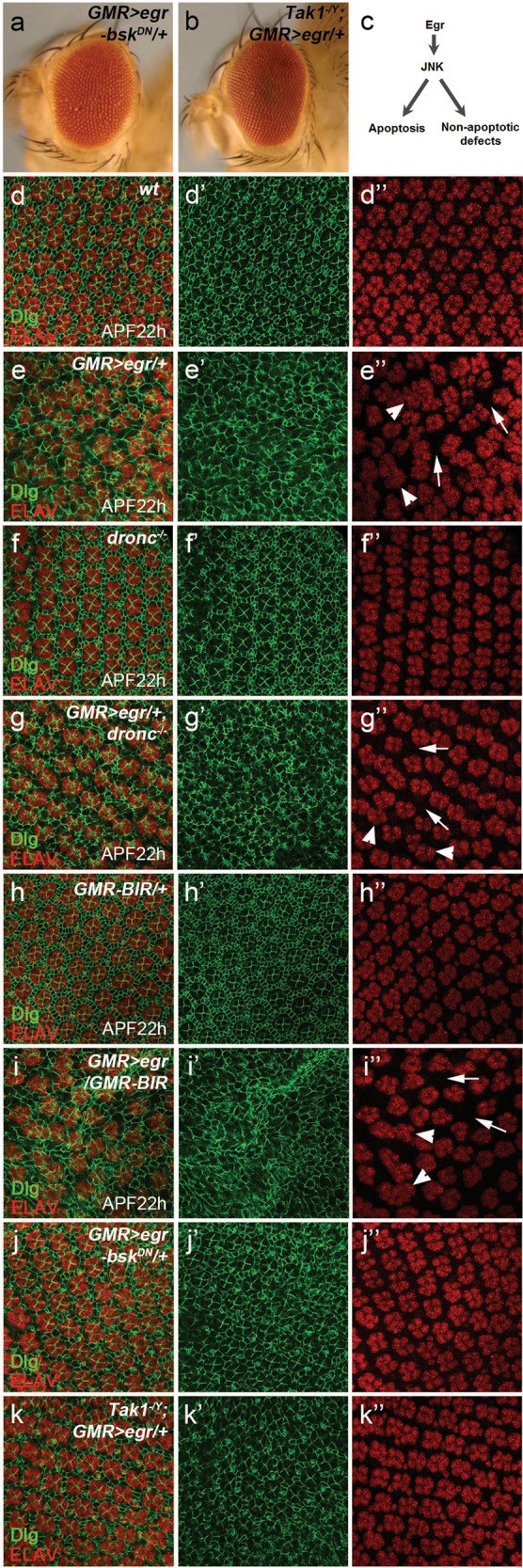
GMR > egr induces nonapoptotic, but JNK-dependent, cellular disorganization in the *Drosophila* eye. **a**, **b** Adult eye images. Expression of a dominant negative form of *bsk* (*bsk*^*DN*^, *bsk* *=* *Drosophila JNK*) (**a**) or a hemizygous mutant of *Tak1* (*Tak1*^*-/Y*^, **b**), an upstream kinase of JNK, almost completely suppresses *GMR* > *egr*-induced eye ablation phenotype. (**c**) A diagram showing that JNK signaling induced by Egr can lead to both apoptosis and nonapoptotic defects in the developing eye. APF22h pupal eye disks labeled with a cellular membrane maker Dlg (green in **d**–**k** and **d′**–**k′**) and a neuronal marker ELAV (red in **d**–**k** and **d′′**–**k′′**). In wild-type disks (**d**–**d′′**), ommatidia (each is composed of eight photoreceptor neurons), as indicated by ELAV, and interommatidial cells, as indicated by Dlg, are well-patterned. In contrast, defective cellular organization was observed in *GMR* > *egr* disks (**e**–**e′′**). Examples of these defects such as ommatidial fusion (arrowheads in **e′′**, **g′′**, **i′′**) and increased interommatidial spacing (arrows in **e′′**, **g′′**, **i′′**) are highlighted. *dronc* null mutants (**f**–**f′′**, *dronc*^−*/−*^) or expression of a stabilized form of Diap1 (**h**–**h′′**, *GMR-BIR*) neither alter the ommatidial patterning in wild-type eye disks (compare **f**–**f′′** and **h**–**h′′** to **d**–**d′′**) nor suppress the irregular ommatidial organization in *GMR* > *egr* disks (compare **g**–**g′′** and **i**–**i′′** to **e**–**e′′**). In contrast, expression of a dominant-negative form of JNK (*bsk*^*DN*^, **j***–***j′′**) or a hemizygous mutant of *Tak1* (**k**–**k′′**) strongly suppresses the cellular disorganization induced by *GMR* > *egr*

Notably, unlike expression of *puc* (Fig. 2d), loss of *dronc* or expression of Diap1 (*GMR* > *BIR*) does not completely restore the *GMR* > *egr* eyes back to normal. These eyes show glassy appearance suggesting defects in their ommatidial patterning (Fig. 2l, m). In contrast, expression of a dominant negative form of *bsk* (*bsk*^*DN*^, *bsk* *=* *Drosophila JNK*) or hemizygous mutants of *Tak1* (*Tak1*^*2527*^, null mutant^41^), an upstream kinase of JNK, almost completely rescue *GMR* > *egr*-induced adult eye defects including the glassy eye appearance (Fig. 3a, b). Therefore, *GMR* > *egr* also induces apoptosis-independent, but JNK-dependent, developmental defects (Fig. 3c).

**Fig. 4:**
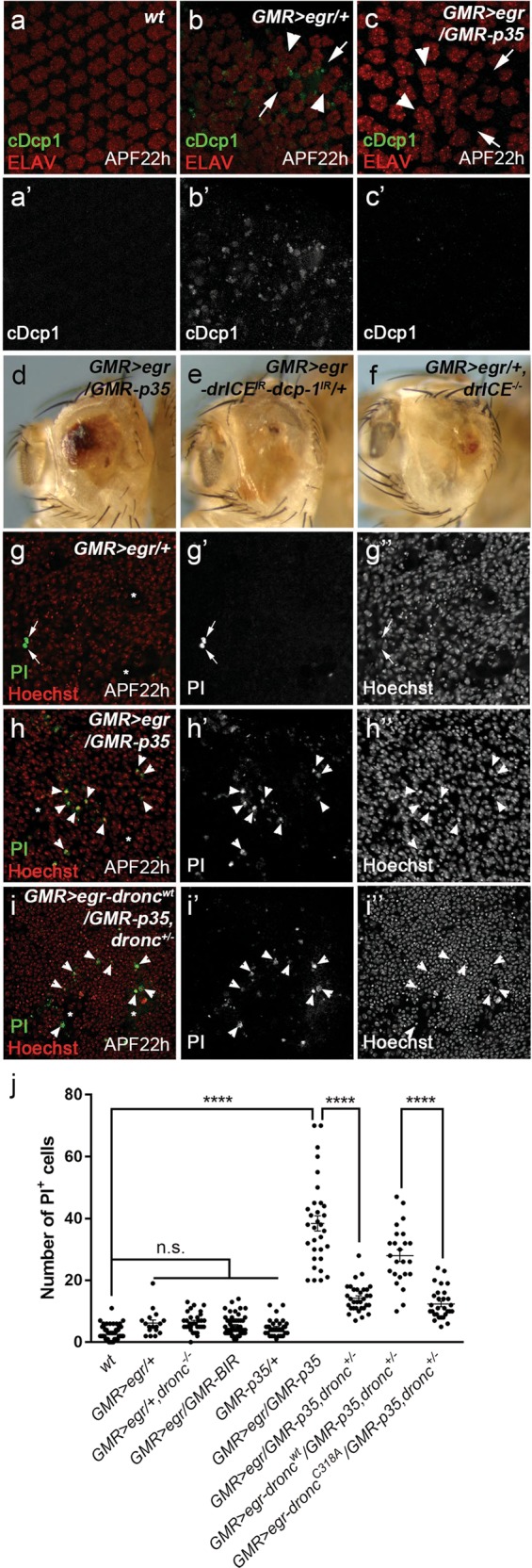
Nonapoptotic cell death is induced in *GMR* > egr when effector caspases DrICE and Dcp-1 are inhibited. APF22h pupal disks labeled with cDcp1 (green in **a**, **b**, **c** and gray in **a′**, **b′**, **c′**) and ELAV (red in **a**, **b**, **c**), a neuronal marker. At this stage, no apoptotic cells were detected in wild-type disks (cDcp1, **a**, **a′**). Ommatidia are also well-patterned (ELAV, **a**). In contrast, strong apoptosis was detected at APF22h in *GMR* > *egr* eye disks (cDcp1, **b** and **b′**). Cellular disorganization indicated by increased interommatidial spacing (arrows, **b**) and ommatidial fusion (arrowheads, **b**) was observed. Although *GMR* > *egr*-induced apoptosis is almost completely blocked by expression of P35 (cDcp1, **c** and **c′**), the irregular ommatidial organization (**c**, arrows and arrowheads) is not suppressed. **d**–**f** Adult eye images. Expression of P35 (*GMR-p35*, **d**), RNAi knockdown of *drICE* and *dcp-1* (**e**), or *drICE* null mutants (**f**) do not or only slightly suppress *GMR* > *egr*-induced eye ablation phenotype (compare 4**d**, **e**, **f** to 2**b**). APF22h pupal eye disks labeled with Propidium Iodide (PI, green in **g**, **h**, **i** and gray in **g′**, **h′**, **i′**) and Hoechst (red in **g**, **h**, **i** and gray in **g′′**, **h′′**, **i′′**). In *GMR* > *egr* disks, PI detects a background level of signals (arrows, **g**–**g′′**) which often do not co-localize with the Hoechst labeling, a nucleus marker. In contrast, expression of P35 (*GMR* > *egr/GMR-p35*) results in a strong increase of PI-positive nuclei, majority of which are also Hoechst-positive (arrowheads, **h**–**h′′**). Suppression of these PI signals in *dronc* heterozygous mutants can be reversed by expression of a wild-type *dronc* transgene (arrowheads, **i**–**i′′**). Asterisks indicate irregular cellular spacing caused by expression of Egr in the corresponding eye disks. **j** Quantification of PI-positive cell numbers in APF22h pupal eye disks of various genetic backgrounds as indicated. One-way ANOVA with Bonferroni multiple comparison test was used to compute *p*-values. Asterisks indicate statistically significant changes (**** *p*< 0.0001). A background low level of PI-labeling was observed in both wild type and *GMR* > *egr* disks. This low PI-labeling in *GMR* > *egr* is not increased in *dronc* mutants or by expression of a stabilized form of Diap-1 (*GMR-BIR*). The PI-labeling is also low in *GMR-p35* disks. In contrast, strong PI-labeling was observed in *GMR* > *egr/GMR-p35* disks. This PI-labeling is largely suppressed in *dronc* heterozygous mutants (*GMR* > *egr/GMR-p35, dronc*^*+/−*^). In this background, further expression of a wild-type form of Dronc (*GMR* > *egr-dronc*^*wt*^*/GMR-p35, dronc*^*+/−*^), but not a catalytic site-mutated form of Dronc (*GMR* > *egr-dronc*^*C318A*^*/GMR-p35, dronc*^*+/−*^), is sufficient to restore the PI signals

To further characterize this nonapoptotic developmental defects, we examined cellular organization in the pupal eye disks, which are composed of well-patterned ommatidia (Fig. 3d, d′′, ELAV labeling) and interommatidial cells (Fig. 3d, d′). At 25 °C, developmental apoptosis occurs at 28 h after pupal formation (APF28h) to remove extra interommatidial cells in eye disks (Supplementary Fig. S2)^42,43^. In contrast, no apoptosis is observed at APF22h (Fig. 4a, a′). We therefore focused our analysis of *GMR* > *egr* on the stage of APF22h to avoid developmental apoptosis. Compared with wild-type eye disks in which ommatidia and interommatidial cells are well-patterned (Fig. 3d–d′′), *GMR* > *egr* induces disorganization of these cells (Fig. 3e–e′′) indicated by both increased interommatidial spacing (Fig. 3e′′, arrows) and ommatidial fusion (Fig. 3e′′, arrowheads). Such defects persist when apoptosis is inhibited in *dronc* mutants (Fig. 3g-g′′) or by expression of Diap1 (Fig. 3i–i′′). As controls, ommatidial organization is not affected by loss of *dronc* (Fig. 3f–f′′) or expression of Diap1 (Fig. 3h–h′′) alone. Moreover, *GMR* > *egr*-induced ommatidial organization defects are strongly suppressed by expression of *bsk*^*DN*^ (Fig. 3j–j′′) or in hemizygous mutants of *Tak1* (Fig. 3k–k′′). Altogether, these data suggest that expression of *egr* induces both apoptosis and nonapoptotic, but JNK-dependent, cellular disorganization in the developing eye (Fig. 3c).

### Inhibition of effector caspases in *GMR* > *egr* induces necrosis

One intriguing observation is that, unlike *dronc* mutants or expression of Diap1, expression of P35, an inhibitor of effector caspases, cannot or only slightly suppresses the *GMR* > *egr*-induced eye ablation phenotype (compare Fig. 4d to Fig. 2b, quantified in Fig. 6f)^18,19,44^. One possible explanation is that P35 may not be sufficient to block *GMR* > *egr*-induced apoptosis because it is a pseudo-substrate of effector caspases^26,45^. We examined this possibility. Compared with wild type (Fig. 4a, a′), *GMR* > *egr* induces massive apoptosis and defective ommatidial patterning in pupal eye disks at APF22h (Fig. 4b, b′). Expression of P35 is sufficient to block apoptosis (Fig. 4c, c′). However, unlike *dronc* mutants or expression of Diap1, expression of P35 in *GMR* > *egr* still results in small adult eyes (compare Fig. 4d to Fig. 2l, m). This is not due to any artefacts induced by P35 itself because double RNAi knockdown of *drICE* and *dcp-1* or *drICE* mutants do not suppress, or even enhance, *GMR* > *egr*-induced eye ablation phenotype (Fig. 4e, f). These RNAi lines and mutants are functional because they can suppress *GMR-hid*-induced apoptosis (Supplementary Fig. S3). These suggest that inhibition of effector caspases, particularly DrICE, in *GMR* > *egr* may induce another form of cell death while it suppresses apoptosis.

**Fig. 5:**
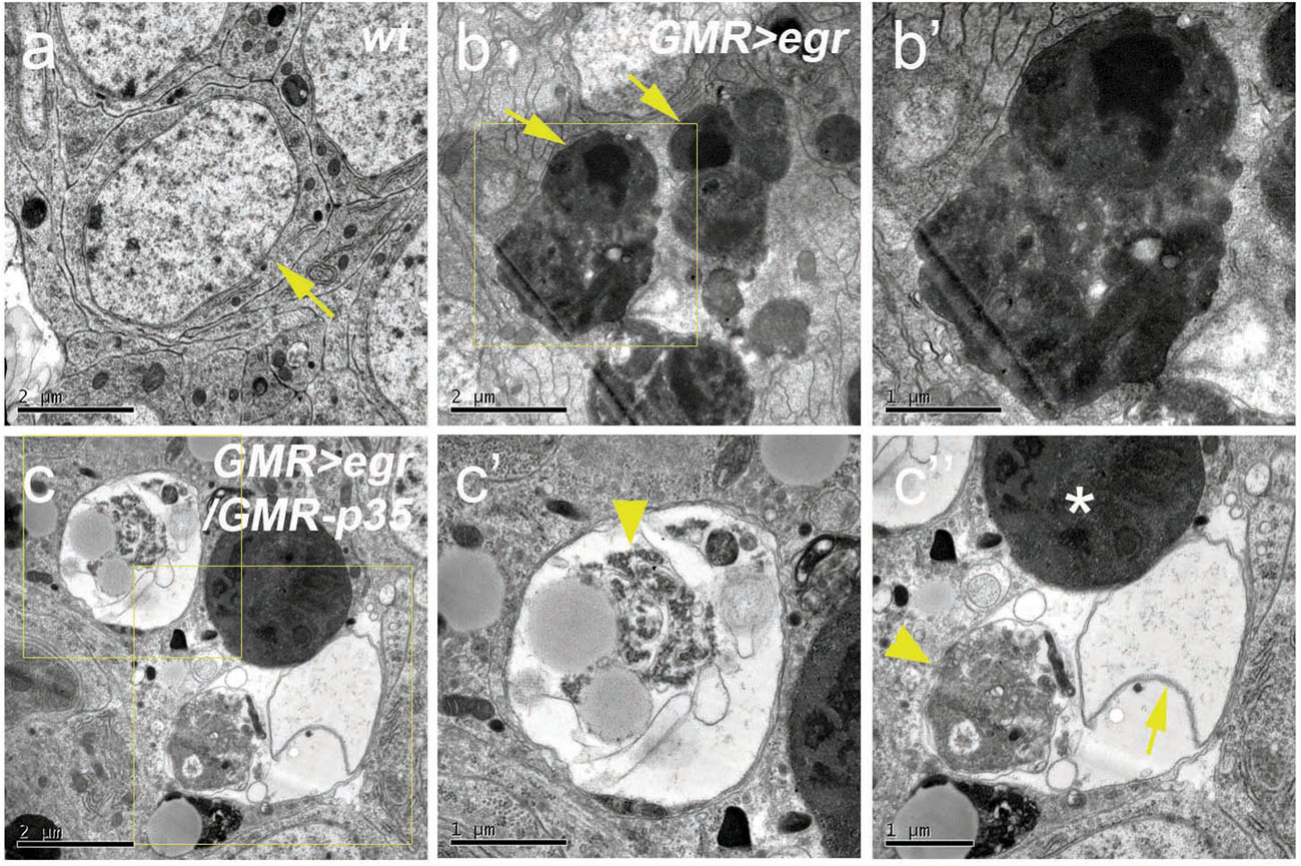
Nonapoptotic cell death in GMR > egr/GMR-p35 shows morphological features of necrosis. TEM images of *wt* (**a**), *GMR* > *egr/* *+* (**b**, **b′**) and *GMR* > *egr/GMR-p35* (**c**–**c′′**) disks at APF22h. **b′** is an enlarged image for the outlined area in **b**. **c′**, **c′′** are enlarged images for the outlined areas in **c**. Compared with the wild-type eye disk cell which has a large nucleus (yellow arrow, **a**), apoptotic features such as high-electron-density chromatin condensation (yellow arrows, **b**) and apoptotic bodies (dark aggregates in **b** and **b′**) are frequently observed in *GMR* > *egr* disks (**b**). Expression of P35 in *GMR* > *egr* (**c**), however, induces necrotic cell features such as translucent cytoplasm, mal-shaped (arrow, **c′′**) or unidentifiable nuclei (**c′**), and aggregation of endoplasmic reticulum and other cellular organelles (arrowheads, **c′** and **c′′**). Asterisk indicates a phagolysosome

To further characterize this nonapoptotic cell death, we performed labeling with propidium iodide (PI), a dye enters a cell and binds to its DNA when the cell membrane is disrupted. Because loss of membrane integrity is a hallmark of necrosis, PI has been used to label necrotic cells (Supplementary Fig. S4a,b)^46,47^. Interestingly, no PI-positive cells were detected in late 3rd instar larval *GMR* > *egr* disks with or without expression of P35 (Supplementary Fig. S4c, d). We then examined a later pupal stage (APF22h). In *GMR* > *egr*, a very low level of PI-labeling was observed (Fig. 4g, g′). Notably, most of these PI signals are unspecific because they do not co-localize with a DNA marker Hoechest (Fig. 4g, g′′, arrows). In contrast, expression of P35 strongly induces PI-labeling in *GMR* > *egr* disks (Fig. 4h, h′). Importantly, majority of these PI signals co-localize with Hoechest-positive nuclei (Fig. 4h, h′′, arrowheads) suggesting that they specifically label membrane-compromised cells. As a control, expression of P35 alone does not induce PI-labeling (Fig. 4j). Altogether, these data suggest that Egr may induce necrosis when apoptosis is blocked. To further confirm this, we performed the Transmission Electron Microscopy (TEM) analysis on *GMR* > *egr/GMR-p35* pupal eye disks. Compared with the wild-type control (Fig. 5a), cells with typical apoptotic features^48^ such as high-electron-density chromatin condensation and apoptotic bodies were frequently observed in *GMR* > *egr* disks (Fig. 5b, b′). In contrast, cells with typical necrotic features were observed in *GMR* > *egr/GMR-p35* disks (Fig. 5c-c′′). These cells have translucent cytoplasm, mal-shaped or unidentifiable nuclei, and aggregation of cellular organelles such as endoplasmic reticulum, which are characteristics of necrotic cell death^48,49^. Therefore, necrosis is induced in *GMR* > *egr/GMR-p35* pupal eye disks.

### The initiator caspase Dronc mediates Eiger-induced necrosis

**Fig. 6:**
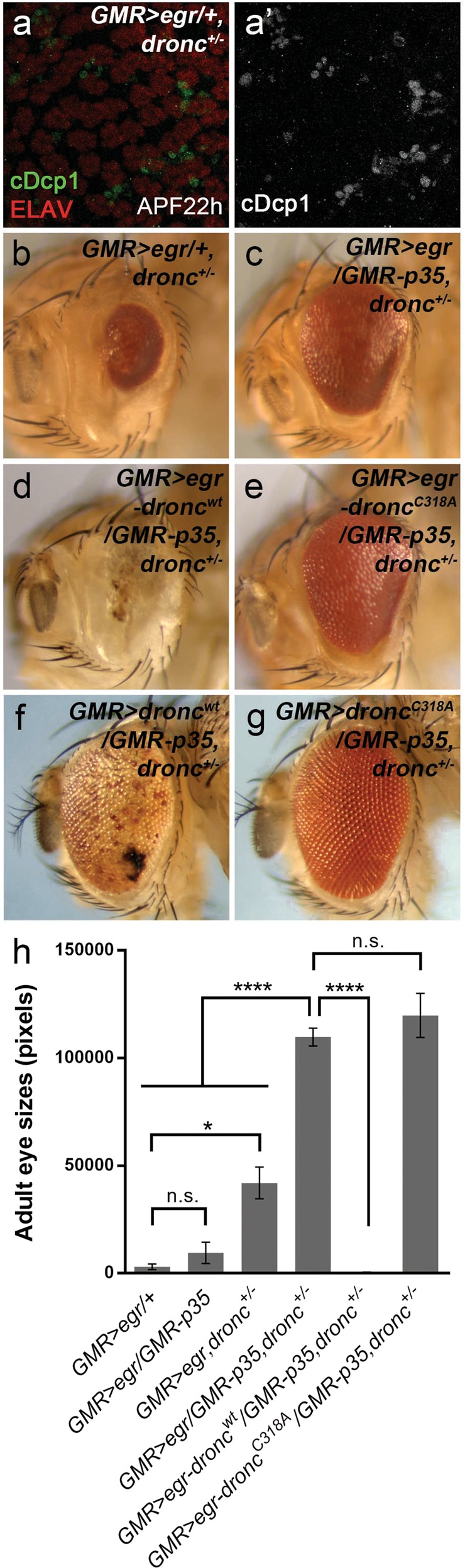
GMR > egr induces Dronc-dependent necrosis when DrICE and Dcp-1 are inhibited. **a**, **a′** APF22h pupal eye disks labeled with cDcp1 (green in **d** and gray in **d′**) and ELAV (red in **d**). Loss of one copy of *dronc* does not or only slightly suppress *GMR* > *egr*-induced apoptosis. **b**–**e** Adult eye images. Although loss of one copy of *dronc* only slightly inhibits *GMR* > *egr*-induced small eye phenotype (compare **6b** to **2b**), it strongly suppresses the eye ablation phenotype induced by *GMR* > *egr/GMR-p35* (compare **6c** to **4d**). This suppression is neutralized by expression of a wild-type form of Dronc (**d**), but not a catalytic site-mutated form of Dronc (**e**). **f**, **g** Adult eye images. Expression of Dronc and P35 in *dronc* heterozygous mutants does not reduce the eye size although it causes an eye pigmentation defect (**f**). Expression of a catalytic site-mutated form of Dronc does not result in any eye defects (**g**). **h** Quantification of the average adult eye size (mean ± SD) of various genetic backgrounds as indicated. One-way ANOVA with Bonferroni multiple comparison test was used to compute *p*-values. Asterisks indicate statistically significant changes (*P < 0.05 or ****P < 0.0001). Suppression of *GMR* > *egr* by expression of P35 is not statistically significant (n.s.). Heterozygous *dronc* mutants only weakly suppress *GMR* > *egr*-induced small eyes (*GMR* > *egr/+, dronc*^*+/−*^). But they strongly suppress *GMR* > *egr/GMR-p35*-induced eye ablation phenotype (*GMR* *>* *egr/GMR-p35, dronc*^*+/*−^). In this background, further expression of a wild-type form of Dronc (*GMR* > *egr-dronc*^*wt*^*/GMR-p35, dronc*^*+/−*^), but not a catalytic site-mutated form of Dronc (*GMR* > *egr-dronc*^*C318A*^*/GMR-p35, dronc*^*+/−*^), is sufficient to restore the eye ablation phenotype

To determine how necrosis is activated when effector caspases are inhibited, we first examined PI-labeling in *GMR* > *egr* disks in a background of *dronc* mutants or with expression of the stabilized Diap1 (*GMR-BIR*). This is because both of them suppress *GMR* > *egr*-induced apoptosis upstream of effector caspases (Fig. 2l, m). Only a background level of PI-labeling was detected under these conditions (Fig. 4j). Strikingly, loss of one copy of *dronc* strongly suppresses the PI-labeling induced in *GMR* > *egr/GMR-p35* (Fig. 4j). However, it only weakly affects *GMR* > *egr*-induced apoptosis (Fig. 6a, a′) hence the eye ablation phenotype (Fig. 6b, h). Consistent with these observations, *dronc* heterozygous mutants strongly suppress the *GMR* > *egr/GMR-p35* small eyes (Fig. 6c, h). Therefore, Dronc is a key component mediating necrosis when *GMR* > *egr*-induced apoptosis is inhibited.

It is known that Dronc, the initiator caspase, cleaves its downstream effector caspases, e.g., DrICE and Dcp-1, to activate apoptosis in *Drosophila*^33,50^. We thus examined whether the catalytic activity of Dronc is required for necrosis induced in *GMR* > *egr/GMR-p35*. To do this, either a wild-type Dronc (*dronc*^*wt*^) or a catalytic site-mutated form of Dronc (*dronc*^*C318A*^) transgene was used^51^. As expected, expression of the wild-type Dronc in *GMR* > *egr/GMR-p35; dronc*^*+/−*^ animals restores PI-positive necrosis (Fig. 4i–i′′, quantified in 4j) and, consequently, the eye ablation phenotype (Fig. 6d, h). In contrast, expression of *dronc*^*C318A*^ in the same background does not have such effects (Figs. 4i and 6e, h). As controls, expression of *dronc*^*wt*^ or *dronc*^*C318A*^ in a similar background without expression of *egr* does not result in small eyes (Fig. 6f, g). Notably, an eye pigmentation defect was observed when *dronc*^*wt*^ is overexpressed (Fig. 6f), consistent with what has been previously reported^33,50^. Altogether, these data suggest that the catalytic activity of Dronc is required for Egr-induced necrosis.

### JNK signaling is required for Eiger- and Dronc-induced necrosis

Unlike *GMR* > *egr*, the eye ablation phenotype induced by expression of *hid*, e.g., *GMR-hid*, can be suppressed by expression of P35 (ref.^26^) or reduction of DrICE and Dcp-1 (Supplementary Fig. S3). Therefore, factor(s) other than inhibition of apoptosis is required for induction of necrosis in *GMR* > *egr/GMR-p35*. Because Egr activates the JNK signaling upstream of *hid*-mediated apoptosis, we examined whether there is a nonapoptotic input from JNK contributing to induction of necrosis when apoptosis is blocked. Hypomorphic mutants of *bsk*, *hep*, *MKK4* and *Tak1*, genes encoding various kinases in the JNK pathway^52^, were used. We observed that heterozygosity of these mutants can only moderately suppress the *GMR* > *egr*-induced eye ablation phenotype (compare Fig. 7a–d to Fig. 2b). In contrast, except *hep*^1^ (Fig. 7f), heterozygosity of *bsk*^1^, *MKK4*^*G680*^ or *Tak1*^2^ mutants strongly suppresses the *GMR* > *egr/GMR-p35* small eyes (compare Fig. 7e, g, h to Fig. 2b). Moreover, we observed that overexpression of Dronc in eye disks, i.e., *GMR* > *dronc*^*wt*^, induces a moderate level of apoptosis (Supplementary Fig. S5a–c). It activates necrosis when apoptosis is blocked (compare Fig. 7j to 7i). In contrast, expression of the catalytic site-mutated form of Dronc does not induce necrosis (Fig. 7k). Because JNK is activated at a basal level in photoreceptor neurons during eye development^53^, we examined whether JNK is required for Dronc-induced necrosis. Although JNK activity is not further enhanced by overexpression of Dronc (Supplementary Fig. S5d–i), loss of one copy of *bsk* or *Tak1*, as well as the *Tak1* null mutants, suppress necrosis in the *GMR* > *dronc*^*wt*^*/GMR-p35* disks (Fig. 7l, m). Therefore, in addition to its roles in apoptosis and development, JNK signaling also contributes to induction of necrosis (Fig. 7n).

**Figure:**
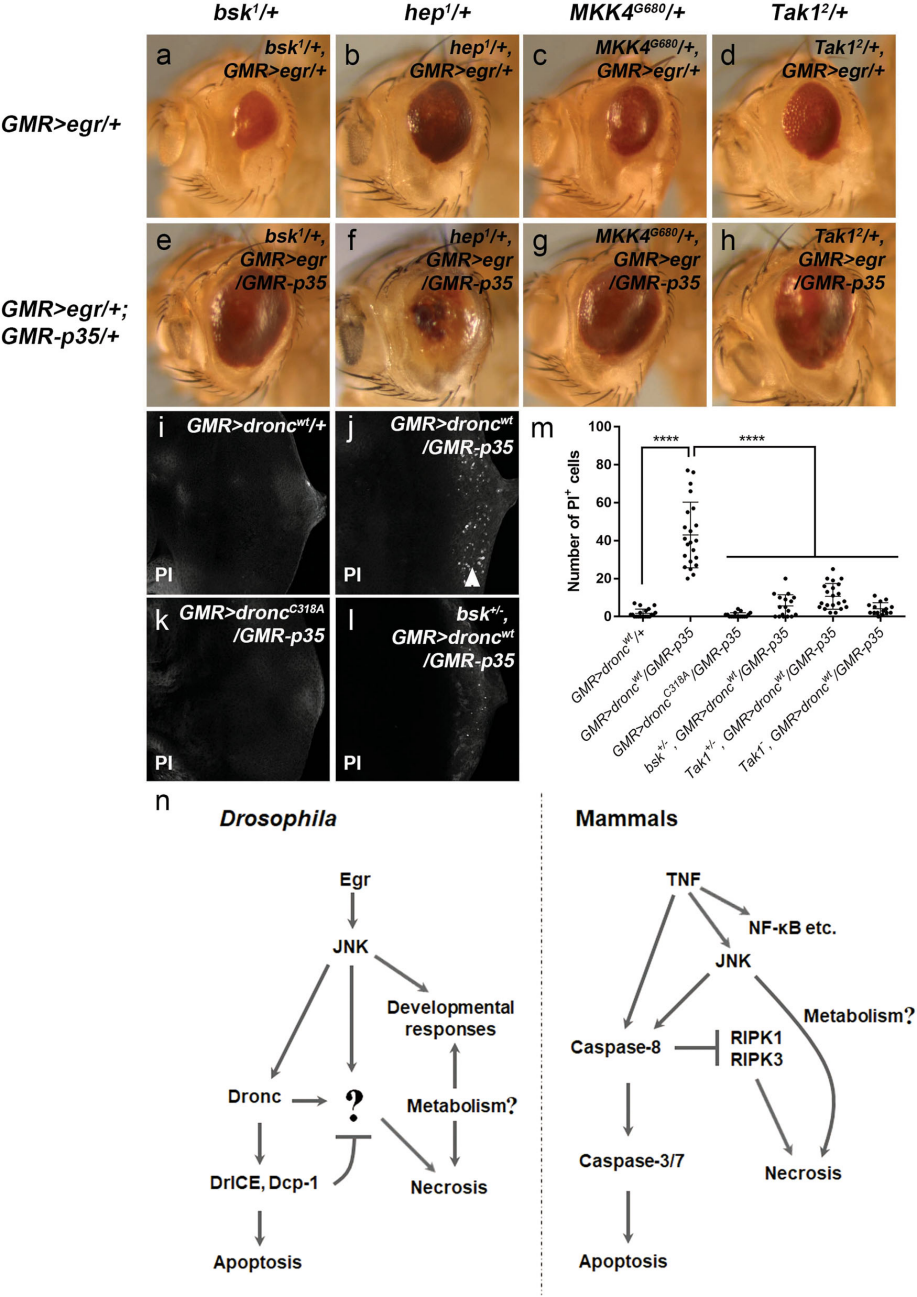
Fig. 7. JNK signaling contributes to Eiger- and Dronc-induced necrosis when apoptosis is blocked. **a**–**h** Adult eye images. Heterozygous mutants of *bsk*^1^ (**a**), *hep*^1^ (**b**), *MKK4*^*G680*^ (**c**) and *Tak1*^2^ (**d**) can only weakly or moderately inhibit *GMR* > *egr*-induced eye ablation phenotype (compared with Fig. 2b). In contrast, *GMR* > *egr/GMR-p35*-induced small eyes are strongly suppressed by heterozygous mutants of *bsk*^1^(**e**), *MKK4*^*G680*^ (**g**) and *Tak1*^2^ (**h**), but not *hep*^1^(**f**) (compared with Fig. 4d). **i**–**l** Late 3rd instar larval eye disks labeled with Propidium Iodide (PI). Compared with expression of a wild-type form of Dronc (*GMR* *>* *dronc*^*wt*^*/*+, **i**), co-expression of Dronc and P35 (*GMR* > *dronc/GMR-p35*) induces PI-positive necrosis (arrowhead, **j**). In contrast, PI-labeling is not observed when a catalytic site-mutated form of Dronc is expressed instead (*GMR* *>* *dronc*^*C318A*^*/GMR-p35*, **k**). Loss of one copy of *bsk* (*bsk*^*+/*−^) strongly suppresses necrosis induced in *GMR* > *dronc*^*wt*^*/GMR-p35* (**l**). **m** Quantification of PI-positive cell numbers in late 3rd instar larval eye disks of various genetic backgrounds as indicated. One-way ANOVA with Bonferroni multiple comparison test was used to compute *p*-values. Asterisks indicate statistically significant changes (**** *p*< 0.0001). Loss of one copy of *Tak1* (*Tak1*^*+/−*^) or a *Tak1* null mutant (*Tak1*^*−/−*^) strongly suppresses necrosis induced in *GMR* > *dronc*^*wt*^*/GMR-p35*. **n** A diagram showing comparable molecular mechanisms of regulated necrosis in *Drosophila* and mammals. The *Drosophila* TNF (Egr), similar to its mammalian counterparts, has multiple context-dependent functions including induction of necrosis when apoptosis is blocked. In mammals, necrosis can occur when inhibition of caspase-8 on RIPK1 and RIPK3 is removed. JNK contributes to both apoptosis and necrosis. While in *Drosophila*, effector caspases DrICE and Dcp-1 inhibit Egr-induced necrosis. Once this inhibition is removed, the initiator caspase Dronc can activate necrosis with an additional input(s) from JNK signaling. Key factors that mediate this necrosis downstream of caspases and JNK are currently unknown (indicated by the question mark). Moreover, energy metabolism regulators have been implicated in regulation of Egr/TNF-induced signaling responses although their exact roles remain to be determined (see “Discussion”)

### Egr/JNK-mediated necrosis restricts the oncogenic potential of *scrib* mutant cells

**Fig. 8:**
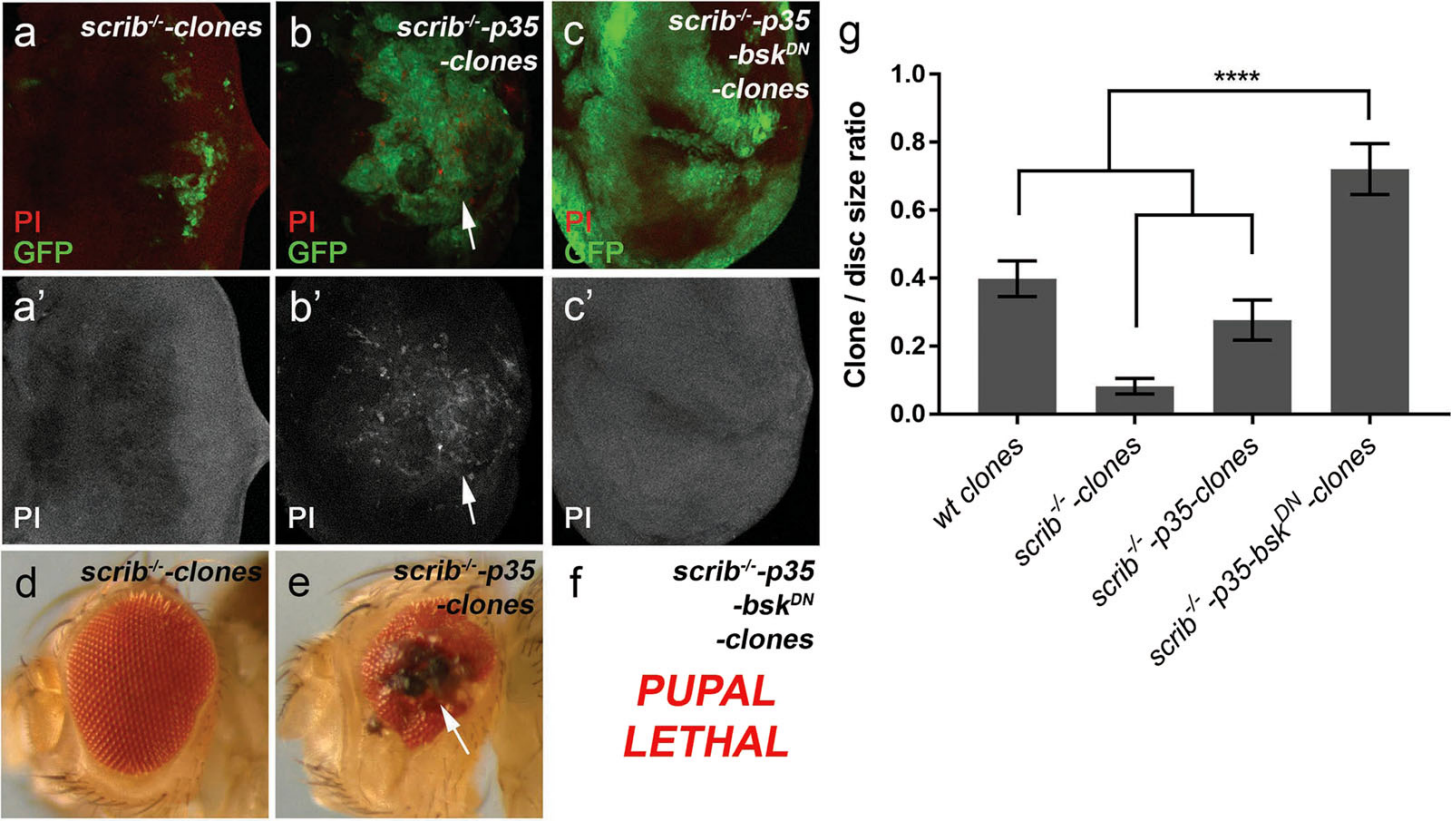
Egr/JNK-mediated necrosis restricts oncogenic growth of *scrib* mutant cells. **a**–**c′** Late 3rd instar larval eye disks with GFP-positive *scrib* mutant (*scrib*^*−/−*^, **a**), *scrib*^*−/−*^*-p35* (**b**) or *scrib*^*−/−*^*-p35-bsk*^*DN*^ (**c**) clones in an otherwise wild-type background. These disks are labeled with Propidium Iodide (PI, red in **a**–**c** and gray in **a′**–**c′**). Compared with *scrib* mutant clones (**a**, **a′**), PI-positive necrosis is induced in *scrib*^*−/−*^*-p35* clones (arrows, **b**, **b′**). This necrosis is suppressed by inhibition of JNK via expression of *bsk*^*DN*^ (**c**, **c′**). **d**, **e** Adult eye images. *scrib* mutant clones only cause a mild rough-eye phenotype (**d**). In contrast, necrotic patches (arrow, **e**) are observed in over 90% of *scrib*^*−/−*^*-p35* mosaic eyes (*n* = 44). **f** *scrib*^*−/−*^*-p35-bsk*^*DN*^ mosaic animals are pupal lethal. **g** Quantification of the clone/disk size ratio in late 3rd instar larval eye disks with various genetic backgrounds as indicated. The ratio is a comparison of the total clone size in each eye disk to the full disk size. One-way ANOVA with Bonferroni multiple comparison test was used to compute *p*-values. Asterisks indicate statistically significant changes (****P < 0.0001). Compared with wild-type clones which occupy an average of 40% of the whole eye disk, *scrib* mutant (*scrib*^*−/−*^) clones are much smaller with an average of 8% coverage on the disk. Expression of P35 in *scrib*^*−/−*^ clones (*scrib*^*−/−*^*-p35*) moderately increases their sizes leading to an average disk coverage of 28%. Further expression of *bsk*^*DN*^ in these clones results in their massive overgrowth and increases the clone/disk ratio to an average of 78%

To further determine roles of Egr/JNK-mediated necrosis under pathophysiological conditions, we examined mutants of *scrib*, a well-characterized tumor suppressor gene regulating the epithelial cell polarity^54–56^. Loss of *scrib* leads to Egr-mediated activation of JNK (Supplementary Fig. S6)^57,58^. Although animals entirely mutant for *scrib* die at the larval stage with massive tissue overgrowth^54^, *scrib* mutant cells (*scrib*^*−/−*^ clones) in an otherwise wild-type tissue are eliminated via JNK-mediated apoptosis^59^. Consistent with these reports, we observed small *scrib* mutant clones in the wild-type larval eye disks (Fig. 8a). Interestingly, when *p35* is expressed in *scrib*^*−/−*^ clones to inhibit apoptosis (*scrib*^−/−^*-p35* clones), these clones show no signs of overgrowth and their average size is close to the wild-type control (Fig. 8b, g). Therefore, apoptosis is not the only mechanism that restricts the oncogenic potential of *scrib* mutant cells. Compared with the *scrib*^*−/−*^ clones (Fig. 8a′), strong PI-labeling was detected in the *scrib*^−/−^*-p35* clones (Fig. 8b′). This suggests that necrosis is induced in these clones. In support of this, compared with adults with *scrib*^*−/−*^ clones (Fig. 8d), over 90% of survived adults (*n* = 44) with *scrib*^*−/−*^*-p35* clones have eyes with necrotic patches (Fig. 8e). We then examined whether JNK is required for necrosis induced in *scrib*^−/−^*-p35* clones. Expression of *bsk*^*DN*^ in the *scrib*^−/−^*-p35* clones (*scrib*^−/−^*-p35-bsk*^*DN*^ clones) completely suppresses the PI-labeling (Fig. 8c′). This results in massively overgrown clones which occupy most of the entire eye disk in all samples we have examined (*n* > 50, Fig. 8c, g). Consequently, these animals are pupal lethal (Fig. 8f). Collectively, these data suggest that, similar to what we observed in the *GMR* *>* *egr/GMR-p35* disks, Egr/JNK-mediated necrosis is activated in *scrib* mutant cells when apoptosis is deficient. Importantly, in this situation, necrosis constrains the oncogenic tissue overgrowth.

## Discussion

Our findings in this study suggest an analogy between *Drosophila* and mammals in regulation of TNF-induced apoptosis and necrosis (Fig. 7n). Caspases play critical roles in these processes. In mammals, caspase-8, an initiator caspase, exerts a permissive role on TNF-induced apoptosis but an inhibitory role on necrosis. Here, we report that both apoptosis and necrosis induced by Egr, the *Drosophila* TNF, depend on the initiator caspase Dronc. Loss of one copy of *dronc* blocks necrosis but not apoptosis suggesting that different levels of Dronc are required for apoptosis versus necrosis. In contrast to Dronc, effector caspases DrICE and Dcp-1, particularly DrICE, appear to inhibit Egr-induced necrosis. Such inhibition can be released by either expression of the inhibitor P35 or reduction of DrICE and Dcp-1. However, inhibition of DrICE and Dcp-1 alone is not sufficient to trigger a switch from apoptosis to necrosis. It requires a further input from JNK signaling. Interestingly, the mammalian JNK has been implicated in promoting TNF-induced and Caspase-8-mediated apoptosis^60^. It can also contribute to necrosis but the underlying mechanisms remain elusive^61,62^. Therefore, JNK may have an evolutionarily conserved role in connecting activation of apoptosis and, if apoptosis fails, necrosis. Such interlinked apoptosis and necrosis may have pathophysiological implications because many human diseases including cancers are frequently associated with both apoptosis and necrosis^63,64^. In this study, we observed that Egr/JNK-mediated apoptosis and necrosis act as a two-layered defense system to restrict the oncogenic potential of *scrib*, a well-established tumor suppressor gene. It is therefore interesting to see whether apoptosis and necrosis cooperate to prevent tumorigenesis in mammals.

Another intriguing observation in our study is that expression of the full-length wild-type Dronc is sufficient to induce necrosis when apoptosis is blocked. Although the full-length Dronc is enzymatically inactive under physiological conditions, its overexpression triggers a moderate level of apoptosis (Supplementary Fig. S5b), which is probably due to spontaneous activation of the full-length Dronc^50^. Suppression of this apoptosis by P35 results in a switch from apoptosis to necrosis (Fig. 7j). Interestingly, although expression of the proapoptotic genes such as *hid* activates Dronc and apoptosis^33,50^, it does not seem to trigger necrosis when apoptosis is blocked. This suggests that activation of Dronc alone is not sufficient to induce necrosis. It has recently been reported that the subcellular localization of Dronc determines its apoptotic versus nonapoptotic functions^65,66^. Therefore, overexpression of Dronc may allow its protein level in a particular subcellular domain to exceed a threshold thus trigger necrosis. This may not occur in *GMR-hid* because it activates but does not induce expression of Dronc (Supplementary Fig. S5i). Notably, the catalytic activity of Dronc is required for both Dronc- and Egr-induced necrosis. Hence, identification of the nonapoptotic substrates of Dronc may provide critical information to understand how Dronc mediates necrosis.

What are other factors that coordinate the inputs from caspases and JNK to differentiate apoptosis versus necrosis? This is an important question that remains largely open. In mammals, caspase-8 cleaves and inactivates RIPK1 and RIPK3 therefore inhibits TNF-induced necroptosis^67,68^. However, no homologs of RIPK1 and RIPK3 have been reported in *Drosophila*. Notably, RIPK3 mediates TNF-induced necrosis at least partially via its role in regulation of energy metabolism and reactive oxygen species (ROS) production^11^. The concept of metabolic checkpoints has been proposed to describe the complex roles of metabolism in regulation of cell death including necrosis^69^. Intriguingly, a recent study in *Drosophila* also revealed roles of the energy metabolism pathways in regulation of *GMR* > *egr*-induced eye ablation phenotype^44^. As we show in this study, expression of Egr can lead to distinct outcomes including apoptosis, nonapoptotic developmental defects and necrosis in a context dependent manner (Fig. 7n). It is therefore interesting to see whether the metabolism pathways regulate Egr-induced necrosis and, if yes, how they interact with caspases and JNK in this process. Furthermore, necrosis-like cell death in *Drosophila* has also been reported in the stressed larval brains^49^, the developmental male testes^70,71^ and female ovaries^72^. In these forms of necrosis, effector caspases and the catalytic activity of Dronc are not required. Hence, future research is needed to understand how different types of necrosis are regulated at the molecular level. What′s more, a series of intracellular events specific to necrosis, such as mitochondrial dysfunction, ATP depletion and increased cytosolic Ca^2+^, appear to be conserved in multiple organisms^73^. Therefore, studies on various necrosis models in *Drosophila* will likely provide insights into understanding the molecular regulation of necrosis.

## Material and methods

### Drosophila genetics

Genetic crosses for all experiments were reared at 25 °C. *GMR-GAL4 UAS-egr* (*GMR* *>* *egr*)^19^, *GMR-hid*^10^ (ref.^29^), *dcp-1*^*Prev1*^ (ref.^74^), *drICE*^*Δ1*^ (ref.^31^), *dronc*^*I29*^ (ref.^36^), *rpr*^*87*^ (ref.^40^), *XR38* (ref.^39^), *hid*^*05014*^ (ref.^29^)*, GMR-BIR*^37^, *GMR-p35* (ref.^26^), *hid*^*20-10*^*-lacZ*^38^, *rpr*^*XRE*^*-lacZ*^38^, *sev* *>* *Glu*^*Lc*^^46^, *MKK4*^*G680*^ (ref. ^75^)*, UAS-dronc*^*wt*^ and *UAS-dronc*^*C318A*^ (ref.^50^), *UAS-bsk*^*DN*^ (on Chr.III) (ref.^76^), *scrib*^1^ and *scrib*^2^ (refs.^77,78^) were as described. *bsk*^1^, *hep*^1^, *Tak1*^*2527*^, *Tak1*^2^ and *UAS-bsk*^*DN*^ (on Chr.X) were obtained from the Bloomington stock center. *UAS-drICE*^*RNAi*^ and *UAS-dcp-1*^*RNAi*^ were obtained from the NIG-Fly stock center. *UAS-egr*^*RNAi*^ (108814) was obtained from the VDRC stock center.

### Mosaic analysis

For mosaic analysis with *hid* mutant clones, larvae of the following genotype were analyzed at the late 3rd instar larval stage: *ey-FLP/*+*; GMR* > *egr/*+*; FRT80B/hid*^*05014*^ *FRT80B*. For mosaic analyses with wild-type control, *scrib* mutant (*scrib*^*−/−*^), *scrib*^*−/−*^*-p35* or *scrib*^*−/−*^*-p35-bak*^*DN*^ clones, the following genotypes were used: (1) *ey-FLP/*+*; act* > *y*+> *GAL4 UAS-GFP/*+*; FRT82B/FRT82B tub-GAL80*; (2) *ey-FLP/*+*; act* > *y*+*>* *GAL4 UAS-GFP/*+*; FRT82B scrib*^1^*/FRT82B tub-GAL80*; (3) *ey-FLP/+; act* *>* *y*+*>* *GAL4 UAS-GFP/UAS-p35; FRT82B scrib*^1^*/FRT82B tub-GAL80*; (4) *ey-FLP/*+*; act* > *y*+> *GAL4 UAS-GFP/UAS-p35; FRT82B scrib*^1^ *UAS-bak*^*DN*^*/FRT82B tub-GAL80*. Similar mosaic analyses with *scrib*^2^ mutants showed comparable results.

### Immunohistochemistry

Pupal or larval disks were dissected, fixed (with 4% paraformaldehyde for 30 min at room temperature), and then labeled with antibodies using standard protocols as described^30^. Primary antibodies used were rabbit anti-cDcp1 (the cleaved Dcp-1 antibody, 1:500, Cell Signaling), rabbit anti-phospho-JNK (pJNK, 1:200, Calbiochem), rat anti-ELAV, mouse anti-Dlg, mouse anti-β-Gal and mouse anti-MMP1 (all 1:50, DHSB). To generate the rabbit anti-Dronc antibody, the full-length *dronc* cDNA was amplified by PCR with primers F-5′CGGGATCCATGCAGCCGCCGGAGCT3′ and R-5′CGGAATTCCTATTCGTTGAAAAACCCGGGATT3′. It was then subcloned into BamHI and EcoRI sites in pET-28a (Novagen) to produce a His-tagged recombinant protein. The purified recombinant protein was then inject into rabbits to generate a polyclonal antibody. The antisera were subsequently affinity purified. Secondary antibodies were goat Fab fragments conjugated to Alex488, 555, or 647 (all 1:1000) from Molecular Probes.

### Propidium Iodidum (PI), Hoechst labeling and TUNEL

For PI and Hoechst double labeling on pupal disks, freshly dissected disks were incubated in dark with 4 µM PI (Sigma-Aldrich) and 16 µM Hoechst (ThermoFisher) in Schneider's media for 1 h at room temperature. The disks were then fixed with 4% paraformaldehyde for 20 min at room temperature followed by gentle washes as previously described^47^. For PI labeling alone, larval or pupal eye disks were incubated with 15 µM PI for 10 min at room temperature followed by fixation (with 4% paraformaldehyde for 20 min at room temperature) and gentle washes as previously described^46^. PI labeling can also be observed and scored without fixation. For TUNEL (terminal deoxynucleotide transferase-mediated dUTP end labeling), dissected and fixed (with 4% paraformaldehyde for 20 min at room temperature) disks were incubated in 100 mM Na-citrate with 0.1% Triton X-100 for 30 min at 65 °C, followed by detection of dying cells using an in situ cell death detection kit (Roche)^30^.

### Imaging and statistical analysis

Fluorescent eye disk images were taken with either a Zeiss or Leica confocal microscope. Adult fly eye images were taken using a Zeiss stereomicroscope equipped with an AxioCam ICC1 camera. For statistical analysis of PI-labeling (Fig. 4j and 7m), at least 20 pupal or larval disks from each genotype were used for counting the number of PI-positive cells. For quantification of adult eye size (Fig. 6h), the “histogram” function in Adobe Photoshop CS was used to measure ten representative adult eyes of each genotype. For quantification of the clone/disk size ratio in late 3rd instar mosaic eye disks (Fig. 8g), the sizes of GFP-positive clones and the whole eye disk were measured using the “histogram” function in Adobe Photoshop CS before the ratio is calculated for each disk. Ten representative mosaic disks of each genotype were used. For all quantifications, the statistical significance was evaluated through a one-way ANOVA with Bonferroni multiple comparison test using GraphPad Prism.

### Transmission electron microscopy (TEM)

Freshly dissected pupal eye–brain complexes were fixed in 2.5% glutaraldehyde in 0.1 M sodium cacodylate buffer (pH 7.4) for 45 min followed by a secondary fixation of 1% Osmium Tetroxide for 1 h at room temperature. The samples were then washed with the buffer (10 × 5 min) and dehydrated in ascending concentrations of ethanol before they were embedded in epoxy resin. Sections (90 nm) were prepared and stained with uranyl acetate and lead citrate followed by examination using a JEOL 1200EX electron microscope fitted with a tungsten filament. Images were acquired through a GATAN MultiScan camera.

## Supplementary information

Supplemental Figure Legend

Supplemental Figure S1

Supplemental Figure S2

Supplemental Figure S3

Supplemental Figure S4

Supplemental Figure S5

Supplemental Figure S6
