# Supplementary material for: Characterization of TNF-induced cell death in Drosophila reveals caspase- and JNK-dependent necrosis and its role in tumor suppression
Source: Cell Death Dis. 2019 Aug 14;10(8):613. doi: 10.1038/s41419-019-1862-0 (PMC6692325; doi:10.1038/s41419-019-1862-0)
Supplement: Supplementary file 1 — Supplemental Figure Legend [file 41419_2019_1862_MOESM1_ESM.docx]

**Supplemental Figures S1-S6**

**Supplemental Figure S1. *GMR>egr* induces transcription of the pro-apoptotic gene *hid*.**

(a-b’) Expression of *hid-lacZ* (indicated by βGAL, red in a, b and grey in a’, b’), a reporter of transcription of *hid*, and cDcp1 labeling (green in a,b) in late 3^rd^ instar larval discs, anterior is to the left. Compared to the control (a,a’), expression of *hid-lacZ* is moderately induced by *GMR>egr* in the *GMR* domain (yellow outlined) where Egr is expressed (b,b’).

(c) Quantification of *hid* expression levels in the *GMR* domain (yellow outlined, a’ and b’) by normalizing its βGAL signal intensity to the signal intensity in the antenna area (blue outlined, a’ and b’) where *GMR* is not expressed. Increase of *hid* expression in *GMR>egr* is statistically significant (P<0.001, Student’s t-test).

(d-e’) Expression of *rpr-lacZ* (indicated by βGAL, red in d, e and grey in d’, e’), a reporter of *rpr* transcription, and cDcp1 labeling (green in d,e) in late 3^rd^ instar larval discs, anterior is to the left. Compared to the control (d,d’), expression of *rpr-lacZ* does not increase in *GMR>egr* (e,e’).

(f) Quantification of *rpr* expression levels in the *GMR* domain (yellow outlined, d’ and e’) by normalizing its βGAL signal intensity to the signal intensity in the antenna area (blue outlined, d’ and e’) where *GMR* is not expressed. No significant (n.s.) increase of *rpr* expression was detected in *GMR>egr*.

**Supplemental Figure S2. cDcp-1 labels developmental apoptosis.**

Pupal discs at APF28h labeled with cDcp1 (green), TUNEL (red) and ELAV (blue). Strong developmental apoptosis as indicated by cDcp1 and TUNEL occurs at APF28h in wild-type discs (a-a’’). This developmental apoptosis is completely suppressed by expression of P35 (b-b’’).

**Supplemental Figure S3. DrICE and Dcp-1 mediate *GMR-hid*-induced apoptosis.**

Adult eye images. *GMR-hid*-induced small eyes (a), resulted from apoptosis, are suppressed by RNAi knockdown of *drICE* and *dcp-1* (b) or *drICE* null mutants (c).

**Supplemental Figure S4. No PI-labeling was detected in *GMR>egr/GMR-p35* larval eye discs.**

Late 3^rd^ instar larval eye discs labeled with Propidium Iodide (PI). In contrast to *Sev>Glu^LC^* in which necrosis, indicated by PI-positive cells (arrowhead, b), is induced (ref. S1), no PI labeling was detected in wild-type (a), *GMR>egr* (c) or *GMR>egr/GMR-p35* (d) larval eye discs.

**Supplemental Figure S5. Expression of Dronc induces a moderate level of apoptosis but not activation of JNK.**

(a-c) Late 3^rd^ instar larval eye discs labeled with TUNEL. Expression of *GMR-GAL4* alone results in a low level of apoptosis, indicated by TUNEL labeling, in the first and second mitotic waves (arrowheads, a-c). Expression of the wild-type full length Dronc induces additional TUNEL-positive apoptotic cells in the GMR domain (arrow, b). This Dronc-induced apoptosis is suppressed by expression of P35 (c).

(d-f) Late 3^rd^ instar larval eye discs labeled with phospho-JNK (pJNK), an antibody recognizing activated JNK. A basal level of JNK activity was observed in photoreceptor neurons in *GMR-GAL4/GMR-p35* (d) and *GMR>dronc^wt^/GMR-p35* (f). In contrast, expression of Egr (*GMR>egr/GMR-p35*) induces strong activation of JNK in the GMR domain (e).

(g-i) Late 3^rd^ instar larval eye discs labeled with Dronc antibodies. A high level of Dronc is detected in *GMR>dronc^wt^/GMR-p35* (h). In contrast, only a background level of Dronc is observed in *GMR-GAL4/GMR-p35* (g) and *GMR-hid/GMR-p35* (i).

**Supplemental Figure S6. JNK activity is induced in *scrib* mutant cells.**

Late 3^rd^ instar larval eye discs with GFP-positive *scrib* mutant (*scrib^-/-^, a*) or *scrib^-/-^-p35* (b) clones in an otherwise wild-type background. These discs are labeled with GFP (green in a,b and grey in a’,b’), MMP1 (red in a,b and grey in a’’,b’’), a downstream target of the JNK pathway, and ELAV (blue in a,b). Activation of JNK, indicated by MMP1 labeling, is observed in both *scrib^-/-^* or *scrib^-/-^-p35* clones.

**Supplemental References**

S1. Yang Y, Hou L, Li Y, Ni J, Liu L. Neuronal necrosis and spreading death in a Drosophila genetic model. Cell death & disease. 2013;4:e723
