# Supplementary figures and images for: Characterization of TNF-induced cell death in Drosophila reveals caspase- and JNK-dependent necrosis and its role in tumor suppression
Source: Cell Death Dis. 2019 Aug 14;10(8):613. doi: 10.1038/s41419-019-1862-0 (PMC6692325; doi:10.1038/s41419-019-1862-0)

Figure S1

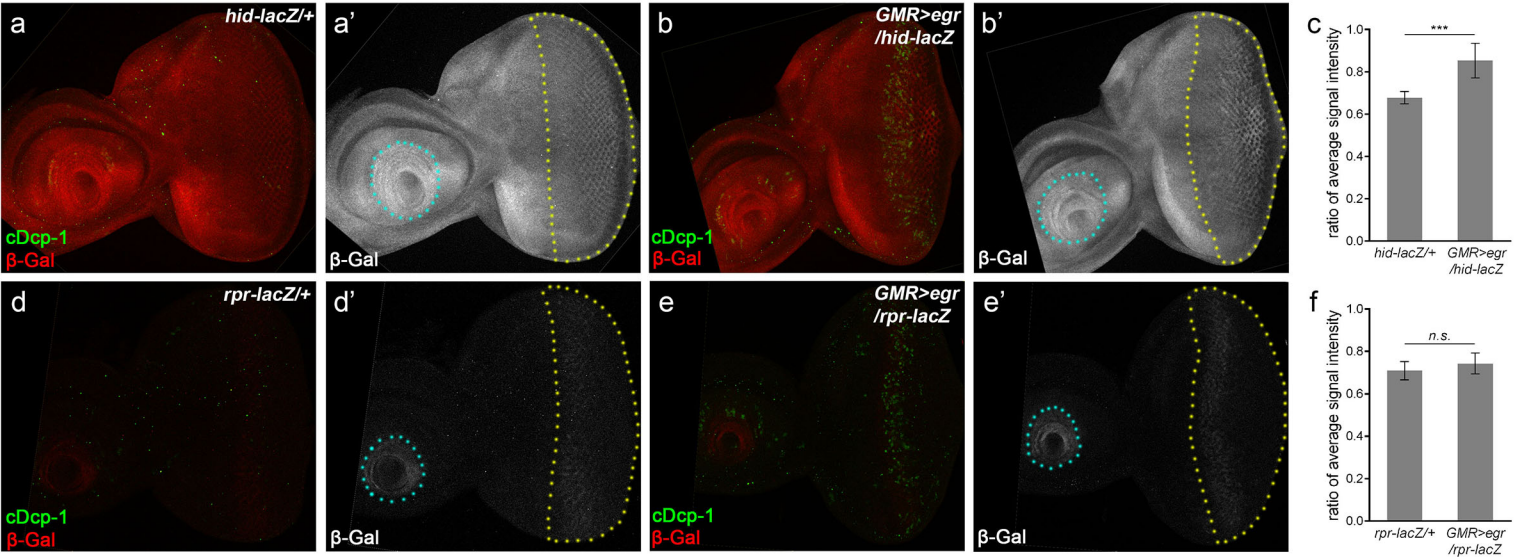

Supplement: Supplementary file 2 — Supplemental Figure S1 [file 41419_2019_1862_MOESM2_ESM.pdf]

Figure S2

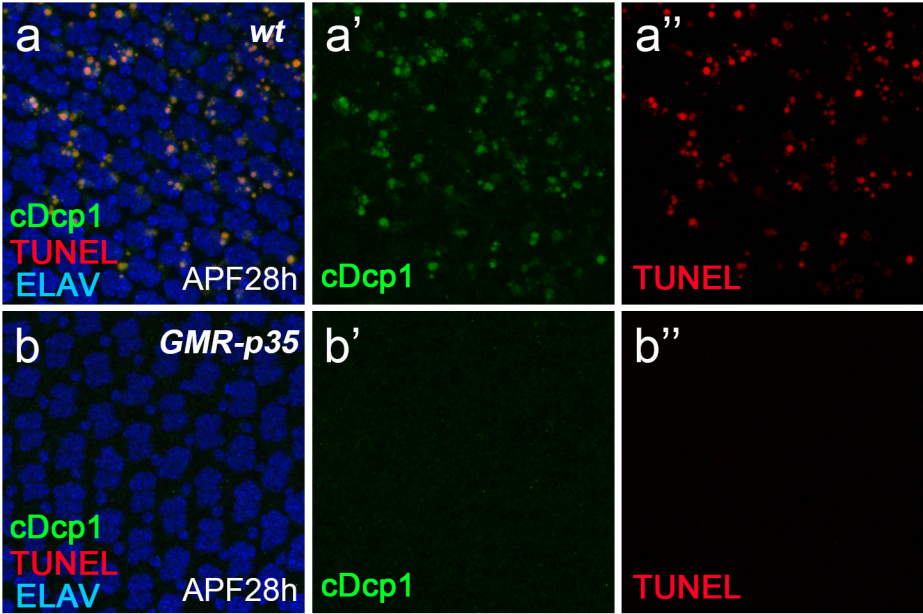

Supplement: Supplementary file 3 — Supplemental Figure S2 [file 41419_2019_1862_MOESM3_ESM.pdf]

Figure S3

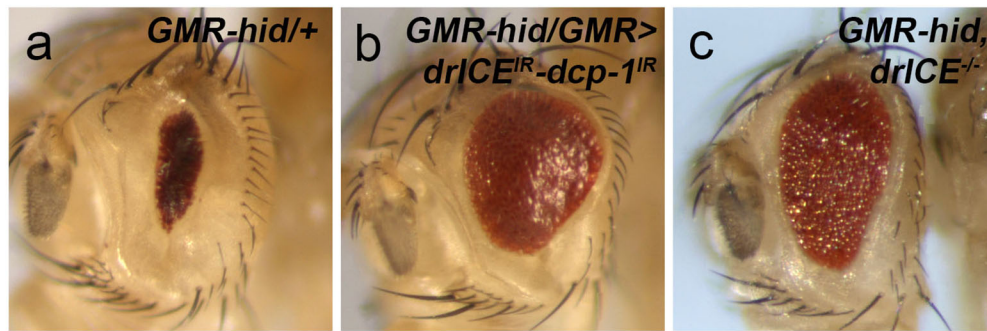

Supplement: Supplementary file 4 — Supplemental Figure S3 [file 41419_2019_1862_MOESM4_ESM.pdf]

Figure S4

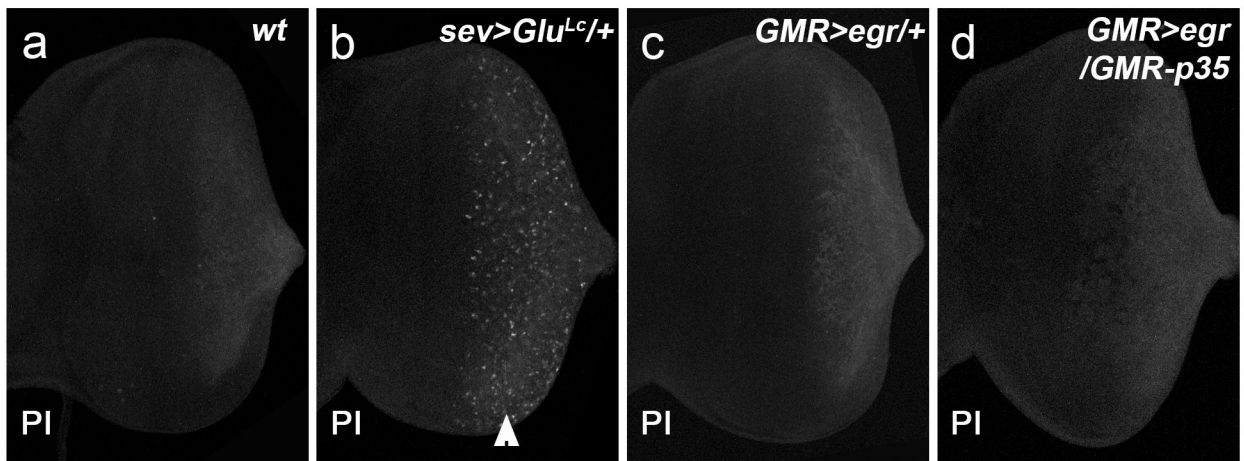

Supplement: Supplementary file 5 — Supplemental Figure S4 [file 41419_2019_1862_MOESM5_ESM.pdf]

Figure S5

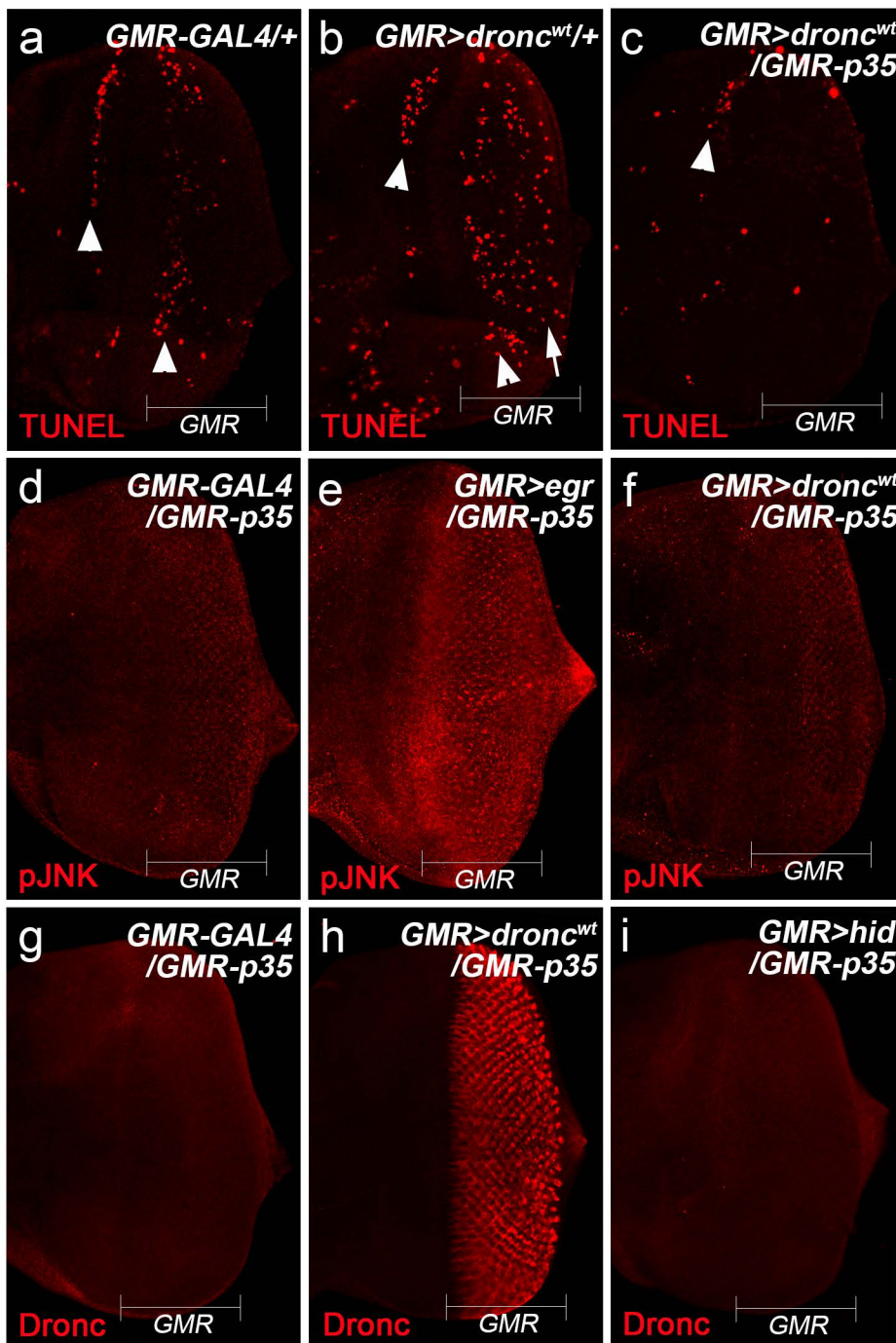

Supplement: Supplementary file 6 — Supplemental Figure S5 [file 41419_2019_1862_MOESM6_ESM.pdf]

Figure S6

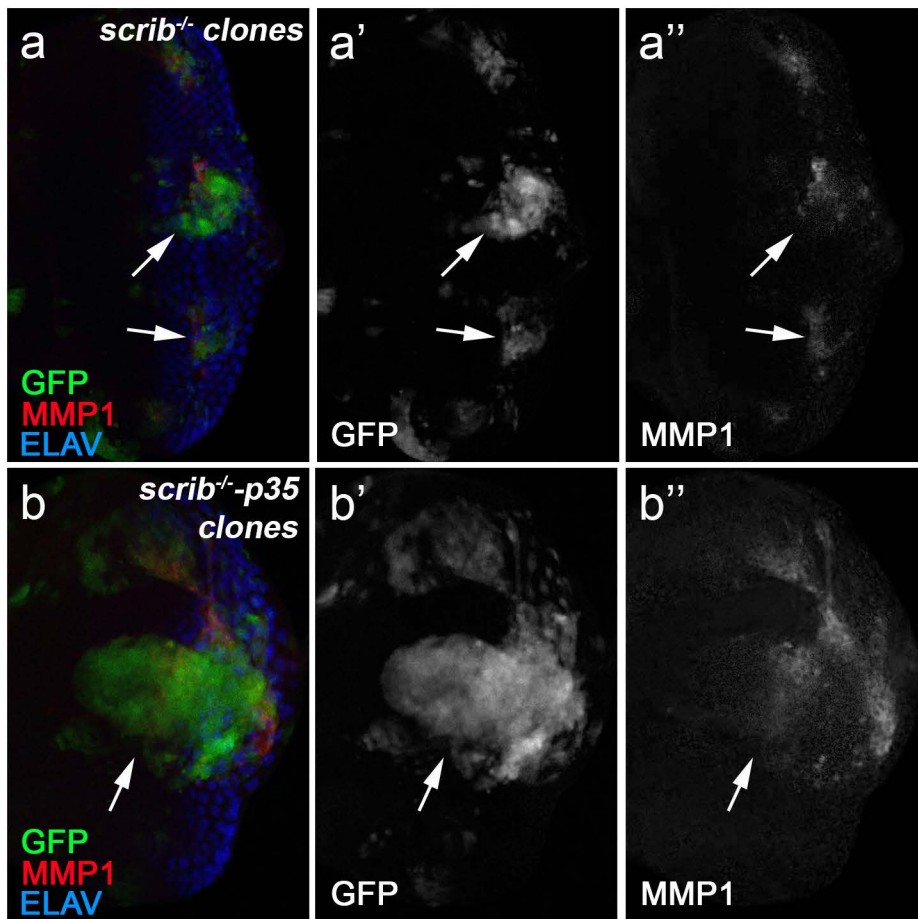

Supplement: Supplementary file 7 — Supplemental Figure S6 [file 41419_2019_1862_MOESM7_ESM.pdf]
